# Evaluating the effectiveness of disaster nursing programs based on Bloom’s taxonomy: a meta-analysis

**DOI:** 10.1186/s12912-025-04266-4

**Published:** 2026-01-08

**Authors:** Seung Ju Lee, Kyeng-Jin Kim

**Affiliations:** 1 College of Nursing, Kyungpook National University, 80 Daehak-ro, Buk-Gu, Daegu, 41944 South Korea; 2 Research Institute of Nursing Innovation, Kyungpook National University, Daegu, Korea, Republic of (South Korea)

**Keywords:** Disaster nursing, Nursing program, Bloom’s taxonomy, Meta-analysis

## Abstract

**Background:**

Effective disaster response demands rapid decision-making and high-quality nursing care. To establish and sustain disaster nursing competencies, structured education and training are indispensable. This study systematically evaluated the effectiveness of disaster nursing programs based on Bloom’s taxonomy of educational objectives.

**Methods:**

A comprehensive literature search was conducted using the keywords “disaster,” “nursing,” “education,” “training,” and “program,” covering publications from January 1, 1900, to August 6, 2024. A total of 12,076 articles were initially identified. After excluding non-interventional studies, books, posters, and studies without calculable effect sizes, 18 articles met the inclusion criteria. Data were analyzed using R version 4.4.2 to compute effect sizes, assess heterogeneity, and evaluate publication bias.

**Results:**

The overall effect size of disaster nursing programs for nurses and nursing students was large (Hedges’ g = 1.12, 95% CI [0.48, 1.77]). By Bloom’s taxonomy domains, the effect sizes were as follows: cognitive domain, g = 0.92 (95% CI [−0.30, 2.14]); affective domain, g = 0.86 (95% CI [0.21, 1.51]); and psychomotor domain, g = 1.19 (95% CI [0.49, 1.89]). For integrated outcomes combining knowledge, attitudes, and skills (other domain), the effect size was g = 0.89 (95% CI [−0.30, 4.08]).

**Conclusion:**

Disaster nursing programs demonstrated the greatest effectiveness in enhancing psychomotor skills, reflecting improvements in practical performance. Strengthening disaster nursing competencies requires comprehensive educational strategies that integrate cognitive, affective, and psychomotor domains while fostering higher-order thinking. These findings highlight the necessity of systematic curriculum design, evidence-based instructional methods, and rigorous evaluation frameworks to advance disaster nursing education.

## Background

Recent climate change and the emergence of novel infectious diseases have made disasters increasingly diverse, complex, and unpredictable. Globally, both the frequency and severity of disasters have risen markedly over recent decades. According to the United Nations Office for Disaster Risk Reduction (UNDRR), between 2000 and 2019, 7,348 major disaster events were recorded, resulting in 1.23 million deaths, affecting 4.2 billion people, and causing an estimated USD 2.97 trillion in global economic losses [1]. The World Health Organization (WHO) underscores that disasters disrupt health systems, cause mass casualties, and generate long-term public health challenges that threaten progress toward universal health coverage and sustainable development [2].

Disasters impose profound strain on healthcare systems. Patient surges frequently exceed hospital capacity, leading to reduced quality of care and increased mortality [3]. Furthermore, physical damage to healthcare infrastructure can interrupt the delivery of essential services [4], while workforce shortages and burnout among healthcare professionals further compromise the sustainability of disaster response efforts [5]. The Sendai Framework for Disaster Risk Reduction 2015–2030, adopted by UN member states in 2015, places human health at the core of disaster risk reduction and calls for strengthened governance, strategic investment in resilience, and enhanced preparedness for effective response [6]. Priority 3 of the framework specifically highlights investment in capacity development among health workers. Complementing this, the WHO Health Emergency and Disaster Risk Management (Health EDRM) Framework (2019) provides a comprehensive structure for mitigating the health risks and consequences of emergencies and disasters [2]. It emphasizes prevention, preparedness, readiness, response, and recovery through ten core components encompassing nearly 200 functions critical for effective disaster risk management, and it highlights the necessity of systematic workforce development and competency strengthening.

Nurses occupy a central role in disaster management owing to their proximity to patients in both time and space [7]. In addition to their clinical responsibilities—such as immediate response, on-site triage, and emergency care—nurses also contribute to disaster planning, policy formulation, and post-disaster evaluation [8]. The International Council of Nurses (ICN), in its Core Competencies in Disaster Nursing Version 2.0 [9], delineates the extensive roles of nurses throughout all disaster phases, recognizing disaster nursing as a specialized discipline that demands complex, systematic expertise extending beyond conventional emergency care [10].

Disaster nursing is defined as the systematic provision of nursing care tailored to individuals affected by disasters across all phases of the disaster cycle [11]. Accumulating evidence demonstrates that disaster nursing programs enhance knowledge, attitudes, and skills [12] and that structured education and training substantially strengthen disaster preparedness among nurses [13, 14]. Such programs encompass the full continuum of disaster management—prevention and mitigation, preparedness, response, and recovery—and may be implemented at the individual, institutional, or community level [15]. Given that required competencies and roles vary across these phases, educational programs designed with clear, domain-specific learning objectives and outcomes are essential for developing comprehensive disaster nursing capacity [16].

While several systematic reviews have explored disaster nursing education, previous investigations have demonstrated notable limitations. Earlier reviews largely focused on general educational outcomes without systematically categorizing findings within established educational frameworks [12]. Some meta-analyses have examined specific instructional modalities, such as simulation-based learning, yet failed to comprehensively evaluate program effectiveness across multiple learning domains [13]. Moreover, no prior meta-analysis has systematically assessed disaster nursing programs using Bloom’s taxonomy as an organizing framework—a critical approach for understanding how such programs influence distinct dimensions of learning: cognitive (knowledge), affective (attitudes and values), and psychomotor (practical skills).

Bloom’s taxonomy of educational objectives provides a structured and comprehensive model for evaluating educational interventions by classifying learning outcomes into three hierarchical domains: cognitive, affective, and psychomotor [17]. The cognitive domain encompasses knowledge acquisition, comprehension, and critical thinking, reflecting the conceptual understanding necessary for effective disaster nursing. The affective domain involves emotions, attitudes, values, and motivation, representing the attitudinal and ethical dimensions of disaster-related practice. The psychomotor domain pertains to motor skills and physical competencies essential for performing clinical procedures and responding effectively in disaster situations [18]. Optimal disaster nursing practice requires the seamless integration of knowledge, technical skills, and preparedness across all three domains to ensure competent and adaptive response [19]. Therefore, comprehensive evaluation of disaster nursing education must systematically encompass these interrelated domains. Understanding the variability in program effectiveness across Bloom’s taxonomy domains holds important implications for both nursing education and disaster management policy.

Accordingly, the present study conducts a meta-analysis to evaluate the effectiveness of disaster nursing education programs within the framework of Bloom’s taxonomy. By identifying which domains are most effectively addressed—and which remain underdeveloped-this analysis aims to provide foundational evidence to inform the design, implementation, and refinement of balanced and outcome-driven disaster nursing curricula.

## Objectives

The specific objectives of this study were as follows:

1. To calculate the effect sizes of disaster nursing programs within the cognitive, affective, and psychomotor domains according to Bloom’s taxonomy and to verify their statistical significance.
2. To examine the heterogeneity of the estimated effect sizes.
3. To assess publication bias to evaluate the validity of the findings.

## Methods

### Design

This study employed a meta-analytic design to systematically and comprehensively evaluate the effectiveness of disaster nursing programs across the cognitive, affective, and psychomotor domains of Bloom’s taxonomy of educational objectives.

### Eligibility criteria

To ensure the comprehensive identification of relevant literature, this review included research articles on disaster nursing programs published in domestic and international journals up to August 6, 2024. Eligible studies were retrieved from January 1, 1900, to August 6, 2024, and were required to apply a disaster nursing program as an intervention. The search strategy combined the following core keywords: “disaster,” “nursing,” “education,” “training,” and “program.” Searches were conducted in both English and Korean using PubMed, the Research Information Sharing Service (RISS), the Korean Studies Information Service System (KISS), and Google Scholar. For each database, search results were displayed 20 entries per page, all pages for each keyword combination were captured, duplicates were removed, and all remaining studies were screened individually.

Studies were included if they met the following criteria: (1) implementation of disaster nursing programs as an intervention; (2) provision of pre- and post-test measurements with specific numerical outcomes for both experimental and control groups; (3) study populations consisting of nurses or nursing students; and (4) publication in English or Korean. Exclusion criteria were as follows: (1) studies lacking sufficient detail regarding program implementation; (2) survey studies, posters, only abstracts, and monographs; and (3) studies for which effect sizes could not be calculated.

**Fig. 1:**
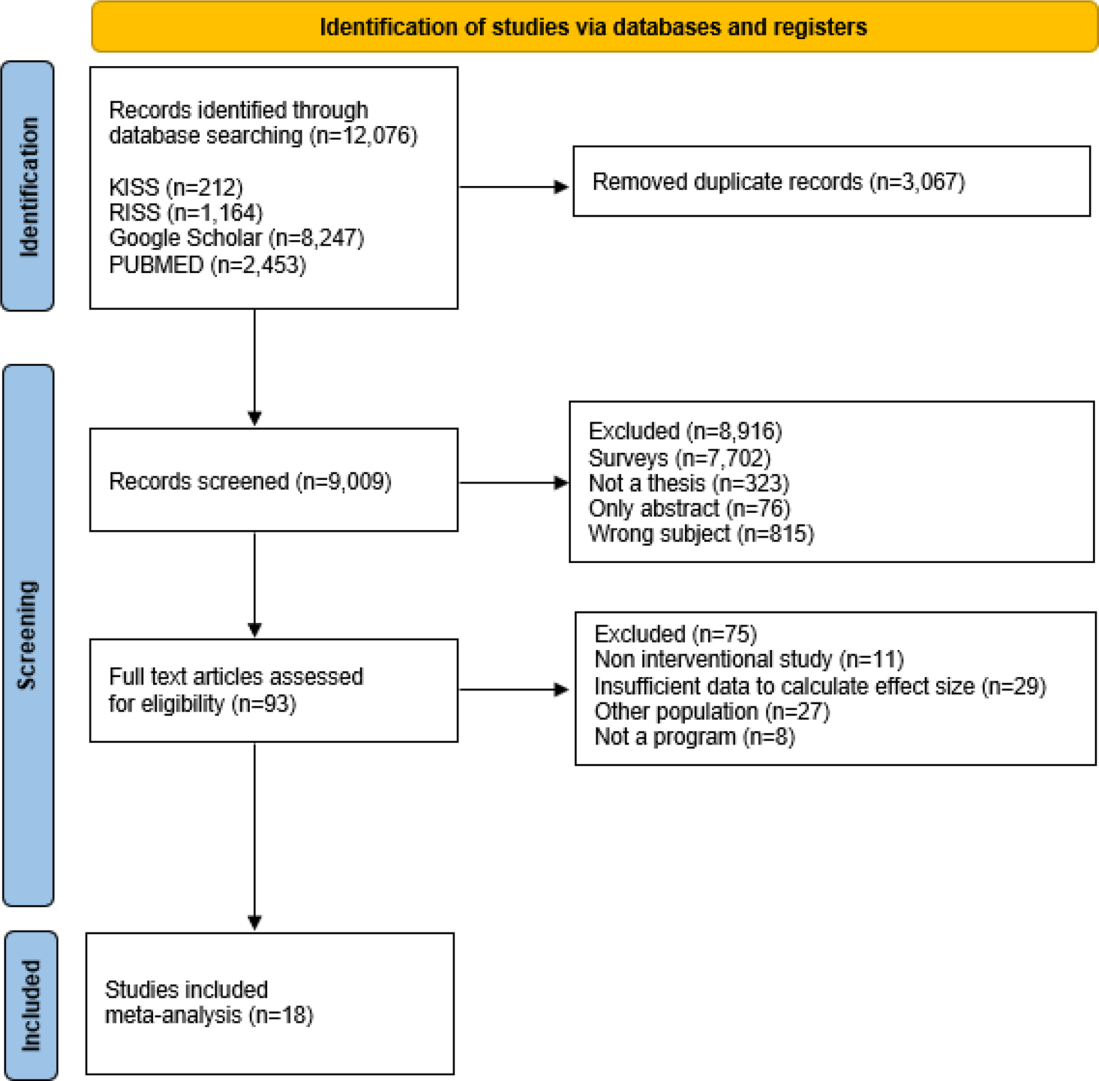
PRISMA study flow chart

This review was conducted in accordance with the Preferred Reporting Items for Systematic Reviews and Meta-Analyses (PRISMA) guidelines. The study selection process is illustrated in Fig. 1.

### Study selection and data extraction

Study selection and data extraction were performed independently by two reviewers. The initial screening was conducted based on study titles and abstracts, followed by a full-text review of all potentially eligible articles. Discrepancies at any stage were resolved through discussion and consensus between reviewers. A standardized data extraction form was used to ensure consistency and completeness. Extracted data included: (1) study characteristics (authors, publication year, country, and design); (2) participant characteristics (sample size and population type); (3) intervention details (type, duration, and delivery method); (4) outcome measures categorized according to Bloom’s taxonomy domains; and (5) statistical data required for effect size calculation (means, standard deviations, and sample sizes for pre- and post-tests in both experimental and control groups). Both reviewers independently extracted all data, and any discrepancies were resolved by re-examining the original studies and reaching consensus.

### Quality assessment

The methodological quality of the 18 included studies was independently evaluated by two reviewers to ensure the validity and reliability of the meta-analytic findings. Appraisal was performed using Cochrane-recommended tools: the Risk of Bias 2.0 (RoB 2.0) tool for randomized controlled trials (RCTs) and the Risk of Bias in Non-randomized Studies of Interventions (ROBINS-I) tool for non-RCTs. The RoB 2.0 assesses bias across five domains: randomization process, deviations from intended interventions, missing outcome data, outcome measurement, and selection of the reported result [20]. The ROBINS-I evaluates potential bias arising from confounding, participant selection, intervention classification, deviations from intended interventions, missing data, outcome measurement, and selective reporting [20]. Before the full assessment, the reviewers jointly conducted a pilot evaluation of two studies to confirm inter-rater reliability and ensure consistency in the application of criteria. Each study was subsequently rated as having a low, moderate, or high risk of bias. Any disagreements were resolved through re-examination of the full text and discussion until consensus was achieved.

### Data analysis

Data from the 18 included studies were extracted regarding program characteristics and study parameters. Pre- and post-intervention means, standard deviations, and sample sizes were coded for both experimental and control groups. Because Cohen’s d may overestimate effect sizes in small samples, corrected effect sizes were computed using Hedges’ g. All meta-analyses were performed using R software (version 4.4.2).

Pooled effect sizes were estimated using a random-effects model to account for expected heterogeneity among studies. Statistical heterogeneity was visually assessed using forest plots and quantified using the I^2^ statistic. Following Higgins’ criteria [21], heterogeneity was considered substantial when I^2^ ≥ 50% and *p* < 0.10 in the test for homogeneity. Publication bias was further evaluated to determine the robustness and validity of the synthesized findings.

### Publication bias assessment

Publication bias was examined using multiple complementary statistical approaches to ensure the robustness of the findings. Egger’s regression test was employed to quantitatively evaluate funnel plot asymmetry by regressing standardized effect sizes against their precision; a statistically significant result (*p* < 0.05) indicated potential publication bias. To further assess robustness, Rosenthal’s fail-safe N statistic was calculated to estimate the number of unpublished studies with null findings required to negate the observed effects. According to Rosenthal’s criterion, a fail-safe N exceeding 5k + 10 (where k is the number of included studies) suggests that the results are resistant to publication bias [22]. In addition, the Trim-and-Fill method was applied to estimate the number of potentially missing studies due to publication bias. After imputing these hypothetical studies, an adjusted overall effect size was recalculated, and publication bias was evaluated by comparing the adjusted and observed pooled estimates.

## Results

### Study selection

The study selection process comprised four stages. A total of 12,076 records were initially identified across all databases. After removing duplicates, 9,009 unique records remained. Title and abstract screening yielded 93 potentially relevant records, and full-text review was conducted for all 93 articles. Of these, 75 full-text articles were excluded, mainly because they were non-interventional studies (*n* = 11), did not provide sufficient data to calculate effect sizes (*n* = 29), involved populations other than nurses or nursing students (*n* = 27), or lacked disaster-related outcomes or relevant program content (*n* = 8). Ultimately, 18 intervention studies met the eligibility criteria and were included in the meta-analysis [23–40] (Fig. 1).

### Characteristics of the included studies

The characteristics of the 18 studies included in this meta-analysis are summarized in Table 1 and detailed in references [23–40]. Regarding study design, eight studies (44.4%) were randomized controlled trials (RCTs) [23, 25–27, 33, 34, 38, 39], and ten (55.6%) were non-randomized controlled trials (non-RCTs) [24, 28–32, 35–37, 40]. Publication years ranged from 2012 to 2024, with the majority of studies (*n* = 15, 83.3%) published after 2018.

**Table 1:** Characteristics of the included studies

	Study				Disaster Nursing Program						Results				
	Author (Year)	RCT	Country	Study population	Program			Program running methods			Domain				Variable
					Program	Frequency	running	Lecture	Practice	Delivery	AD	CD	PD	O	
1 [23]	Ko&Choi.(2024)	RCT	South Korea	N	web browsers (smartphone or PC)	1	100 m	●	●	Online			●		Disaster mental health competence
													●		Problem solving process
											●				Self-leadership
											●				Learning self-efficacy
											●				Motivation to transfer
2 [24]	Ghezeljeh et al.(2018)	N-RCT	Iran	N	Lecture+Telegram application	34	34 Day	●		Online		●			Knowledge
											●				Attitude
3 [25]	Koca&Arkan.(2020)	RCT	Türkiye	NS	LMS, Jennings Disaster Nursing Management Model	N/A	12 Week	●		Online			●		DPPSN Total
													●		DPPSN Preparatory
													●		DPPSN Response
													●		DPPSN Postdisaster
											●				DRSES Total
											●				DRSES On-site
											●				DRSES Psychological
											●				DRSES Role
4 [26]	Kilic&Simsek.(2019)	RCT	Türkiye	NS	Lecture, case studies, abstract tree model, exercises and role-playing et al.	6	6 Week	●	●	Face to Face			●		Perception of prepardness : Preparation Phase
													●		Perception of prepardness : Intervention Phase
													●		Perception of prepardness : Post-disaster Phase
											●				General Self-efficacy
5 [27]	Yildiz&Yildirim.(2022)	RCT	Türkiye	NS	Theoretical+Practice sessions	8	8 Day	●	●	Face to Face	●				Belief in general disaster preparedness scale (BGDPS)
											●				Disaster response self-efficacy scale (DRSES)
											●				Brief resilience scale (BRS)
6 [28]	Kang et al.(2023)	N-RCT	South Korea	NS	Online+Simulation program	3	3 Week	●	●	Mixed		●			COVID-19 Knowledge
											●				COVID-19 nursing Intention
											●				Learning self-efficacy
													●		Clinical performance
7 [29]	Kong et al.(2019)	N-RCT	South Korea	NS	Simulation	1	1 Day	●	●	Face to Face		●			Disaster nursing knowledge
											●				Attitude toward disaster
													●		Basic competency in disaster nursing
8 [30]	Jung et al.(2018)	N-RCT	South Korea	NS	Lecture, conversations, small group learning, practice	8	2 Week	●	●	Face to Face		●			Disaster nursing knowledge
														●	Preparedness
											●				Self-confidence
9 [31]	Kim et al.(2020)	N-RCT	South Korea	N	Tabletop simulation	1	1 Day	●	●	Face to Face		●			Disaster knowledge
												●			Disaster perception
													●		Disaster nursing competency
														●	Disaster preparedness
10 [32]	Aliakbari et al.(2022)	N-RCT	Iran	N	Tabletop, Work shop, operational maneuvers	1	1 Day	●	●	Face to Face			●		Nurse competence
11 [33]	Ma et al.(2021)	RCT	China	NS	Game	1	110 m	●	●	Face to Face			●		Nursing competence
												●			Cognition
													●		Skill
											●				Affective response
12 [34]	Liao et al.(2022)	RCT	China	NS	Simulation	16~	4 Month	●	●	Mixed				●	Disaster Preparedness
											●				Confidence
													●		Performance
13 [35]	Huh&Kang.(2018)	N-RCT	South Korea	NS	Lecture	4	4 Week	●	●	Face to Face		●			Disaster nursing knowledge
													●		Disaster triage
														●	Disaster readiness
14 [36]	Inkaew&Chompunud.(2018)	N-RCT	Thailand	NS	Interactive, Lecture-style teaching method	7	7 Week	●	●	Face to Face			●		Nursing competency (prevention&mitigation)
													●		Nursing competency (preparedness)
													●		Nursing competency (response)
15 [37]	Xia et al.(2019)	N-RCT	China	NS	Interactive-style teaching method	1	1 Day	●	●	Face to Face		●			Disaster fundamentals knowledge
												●			Disaster triage knowledge
												●			Family preparedness knowledge
													●		Disaster fundamentals skill
											●				Disaster fundamentals attitude
													●		Disaster triage skill
											●				Disaster triage attitude
													●		Family preparedness skill
											●				Family preparedness attitude
16 [38]	Farra et al.(2012)	RCT	USA	NS	3-D virtual reality simulation	1	30 m	●	●	Online		●			Knowledge
17 [39]	Fathoni et al.(2019)	RCT	Indonesia	NS	Tabletop disaster exercise	1	1 Day	●	●	Face to Face		●			Knowledge
18 [40]	Lee&Jung.(2023)	N-RCT	South Korea	NS	Tabletop disaster simulation	N/A	N/A	●	●	Mixed		●			Disaster preparedness
													●		Core competency

Nurse = N; Nursing Student = NS; PD = Psychomotor Domain; CD = Cognitive Domain; AD = Affective Domain; O = Others (Knowledge, Attitude, Skills); N/A = Not Available

Geographically, most studies were conducted in South Korea (*n* = 7, 38.9%) [23, 28–31, 35, 40], followed by China (*n* = 3, 16.7%) [33, 34, 37], Türkiye (*n* = 3, 16.7%) [25–27], Iran (*n* = 2, 11.1%) [24, 32], the United States (*n* = 1, 5.6%) [38], Indonesia (*n* = 1, 5.6%) [39], and Thailand (*n* = 1, 5.6%) [36]. Participants were predominantly nursing students (*n* = 14, 77.8%) [25–30, 33–40], while practicing nurses accounted for the remaining four studies (22.2%) [23, 24, 31, 32].

Regarding intervention type, simulation-based education was the most frequently employed approach (*n* = 6, 33.3%) [28, 29, 31, 34, 39, 40], followed by lecture-based education (*n* = 4, 22.2%) [24, 26, 30, 35] and tabletop exercises (*n* = 3, 16.7%) [31, 32, 39]. The majority of studies implemented face-to-face instruction (*n* = 11, 61.1%) [26, 27, 29–33, 35–37, 39], while four studies (22.2%) utilized online delivery [23–25, 38] and three (16.7%) employed mixed delivery methods [28, 34, 40]. Analysis of outcome domains revealed that the cognitive domain was most frequently assessed (*n* = 16, 88.9%), followed by the affective domain (*n* = 11, 61.1%), and other integrated domains encompassing knowledge, attitudes, and skills (*n* = 18, 100%). Only one study (5.6%) explicitly evaluated the psychomotor domain through separate skill assessment.

### Risk of bias in the included studies

**Fig. 2:**
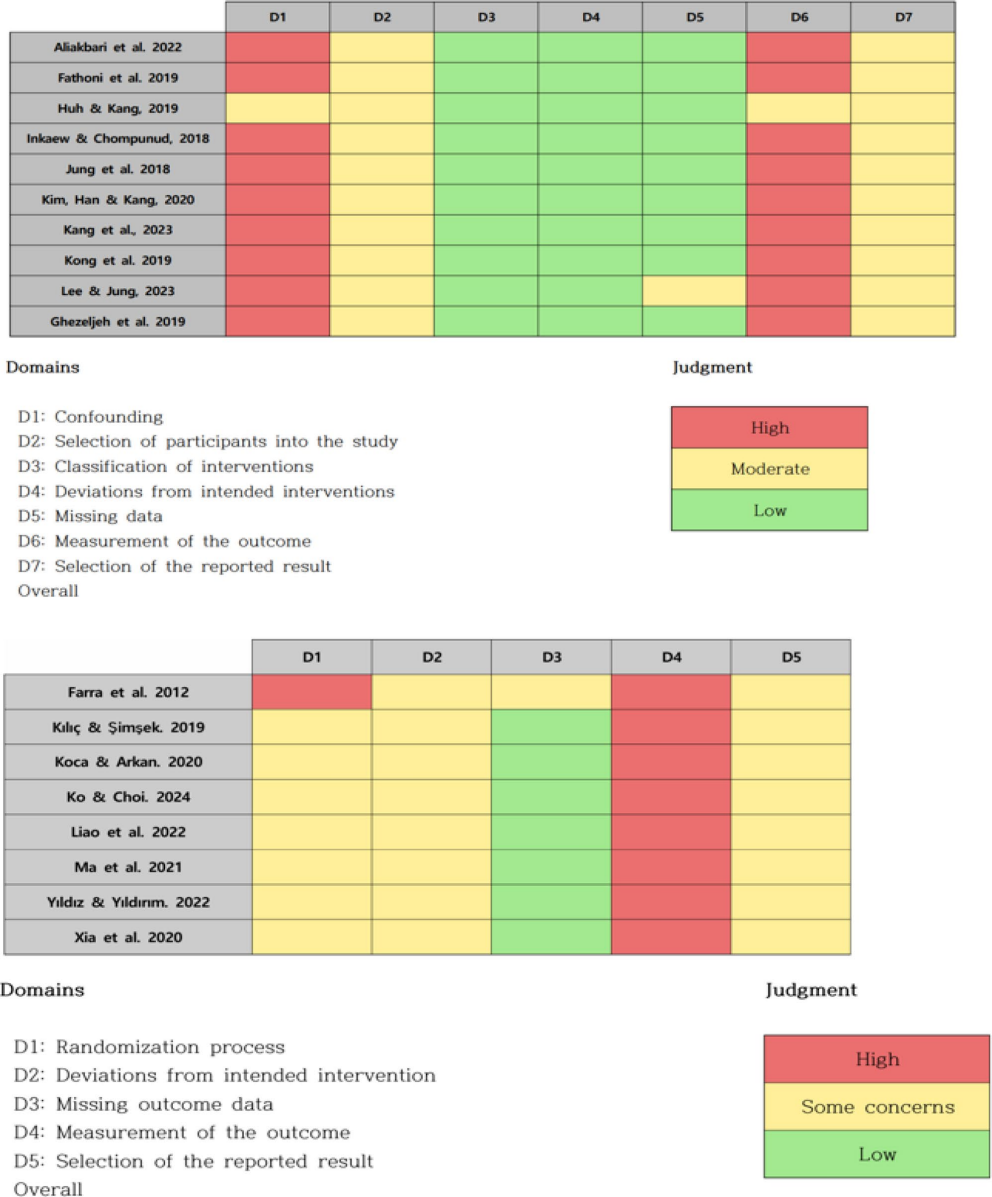
Risk of bias in the included studies

The methodological quality of the 18 included studies is summarized in Fig. 2. Eight RCTs were evaluated using the revised Cochrane Risk of Bias tool (RoB 2.0), and ten non-randomized controlled trials (non-RCTs) were assessed using the Risk of Bias in Non-randomized Studies of Interventions (ROBINS-I) [41].

Among the RCTs, two studies (25%) were judged to have a low risk of bias, five (62.5%) raised some concerns, and one (12.5%) was rated as high risk. Regarding the randomization process, although most studies reported randomization, four (50%) provided insufficient details on sequence generation or allocation concealment, resulting in a rating of “some concerns.” In the domain of deviations from intended interventions, blinding of participants and facilitators was not feasible due to the educational nature of disaster nursing programs. Nevertheless, adherence to intervention protocols was generally high, and analyses were performed as assigned, leading to low risk ratings in five studies (62.5%). For missing outcome data, attrition rates were low, with six studies (75%) reporting dropout rates below 10%, and were therefore rated as low risk. In terms of outcome measurement, studies employing objective assessment tools were judged to have a low risk of bias, whereas three studies (37.5%) relying solely on self-reported measures were rated as having “some concerns.” For selective reporting, five studies (62.5%) were considered low risk, as they had preregistered protocols or comprehensively reported all expected outcomes.

Among the non-RCTs, one study (10%) was rated as low risk, seven (70%) as moderate risk, and two (20%) as high risk. In the domain of confounding, only two studies (20%) adequately controlled for baseline characteristics, while six (60%) did not account for important confounders and were therefore rated as moderate risk. Most studies clearly described eligibility criteria and selected both intervention and control groups from the same source population, resulting in low risk ratings in seven studies (70%) for participant selection. Intervention classification was well defined in the majority of studies, with nine (90%) judged as low risk. Deviations from intended interventions and missing outcome data were generally rated as low risk, except for one study with dropout rates exceeding 20%, which was considered high risk. For outcome measurement, studies employing blinded assessors were rated as low risk, while four (40%) that relied exclusively on self-reported data were rated as moderate risk due to potential measurement bias.

Overall, the methodological quality of the included studies was considered acceptable and appropriate for evidence synthesis.

### Effect sizes of disaster nursing programs

**Fig. 3:**
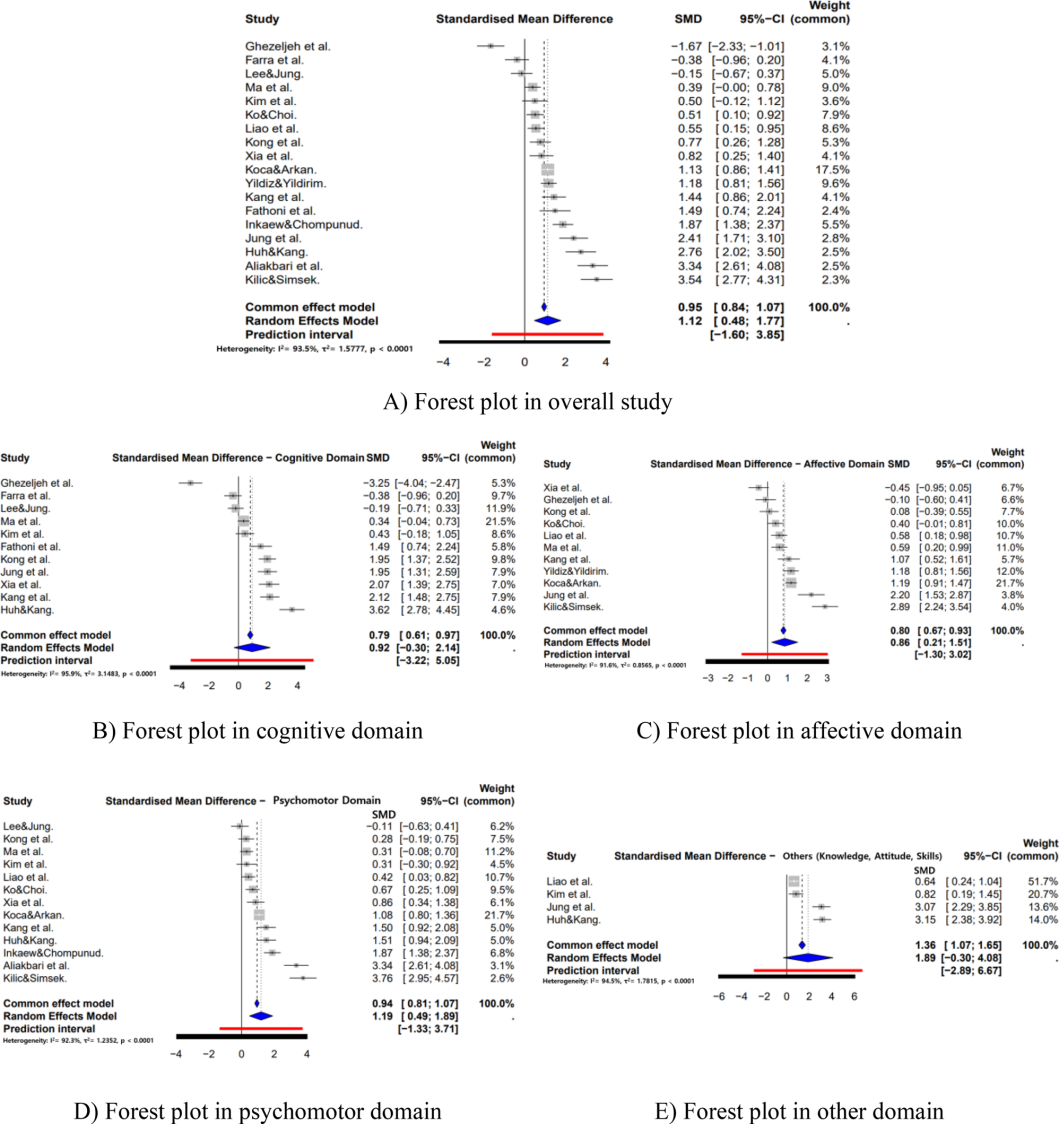
Forest plot of the effects of disaster nursing programs

The overall pooled effect size of disaster nursing programs was large (Hedges’ g = 1.12, 95% CI [0.48, 1.77], *p* < 0.001), according to Cohen’s (1988) criteria [42]. When analyzed by Bloom’s taxonomy domains, the psychomotor domain exhibited the largest effect (Hedges’ g = 1.19, 95% CI [0.49, 1.89], *p* < 0.001). The affective domain demonstrated a large and statistically significant effect (Hedges’ g = 0.86, 95% CI [0.21, 1.51], *p* = 0.010), supported by 11 studies that evaluated self-efficacy, attitudes, and confidence. In contrast, the cognitive domain yielded an effect size of Hedges’ g = 0.92 (95% CI [−0.30, 2.14], *p* = 0.139), which was not statistically significant despite being based on 16 studies examining disaster-related knowledge and preparedness awareness. Across all domains, heterogeneity was substantial, with I^2^ values ranging from 91.6% to 95.9% (Fig. 3).

### Heterogeneity of effect sizes

A total of 26 effect sizes were extracted from the 18 included studies. The pooled effect size remained large (Hedges’ g = 1.12, 95% CI [0.48, 1.77], *p* < 0.001), but substantial heterogeneity was observed across studies (I^2^ = 93.5%, τ^2^ = 1.5777). Domain-specific analyses also indicated very high heterogeneity, with I^2^ values ranging from 91.6% to 95.9%. To further explore potential sources of heterogeneity [43], a meta-ANOVA was conducted using study design, study population, and delivery method as moderator variables (Table 2).

**Table 2:** Effects of moderator variables on program outcomes

Category	Subgroup	K	Hedges’g	95% CI		I^2^ (%)	Q_b_(*p*)
				Low CI	Upper CI		
Study design	Non-RCT	10	1.17	0.352	1.986	94.6	16.22 ( < 0.001)
	RCT	8	0.91	0.309	1.513	93.4	
Study population	Nurse	4	0.82	−0.947	2.592	97.2	16.19 ( < 0.001)
	Nursing student	14	1.12	0.628	1.605	92.7	
Delivery Method	Face-to-Face	11	1.548	0.942	2.155	92.2	15.11 ( < 0.001)
	Online	4	0.085	−0.837	1.007	94.1	
	Mixed	3	0.582	−0.26	1.424	88.5	

K = number of studies; Q_b_ = Q-value between subgroups; Non-RCT = non-randomized controlled trials; RCT = randomized controlled trials; CI = confidence interval

By study design, non-randomized controlled trials (non-RCTs) demonstrated a larger pooled effect size (Hedges’ g = 1.17) compared with RCTs (Hedges’ g = 0.91), with a statistically significant between-group difference (Qb = 16.22, df = 1, *p* < 0.001). Subgroup analysis by study population revealed smaller effect sizes among nurses (Hedges’ g = 0.82) compared with nursing students (Hedges’ g = 1.12). Regarding delivery method, face-to-face education produced the largest effect size (Hedges’ g = 1.55), whereas online education demonstrated a negligible effect (Hedges’ g = 0.09). Mixed-method programs yielded a moderate effect size (Hedges’ g = 0.58), although wide confidence intervals indicated variability in effectiveness. Between-group differences across delivery methods were statistically significant (Qb = 15.11, df = 2, *p* < 0.001).

### Publication bias

**Fig. 4:**
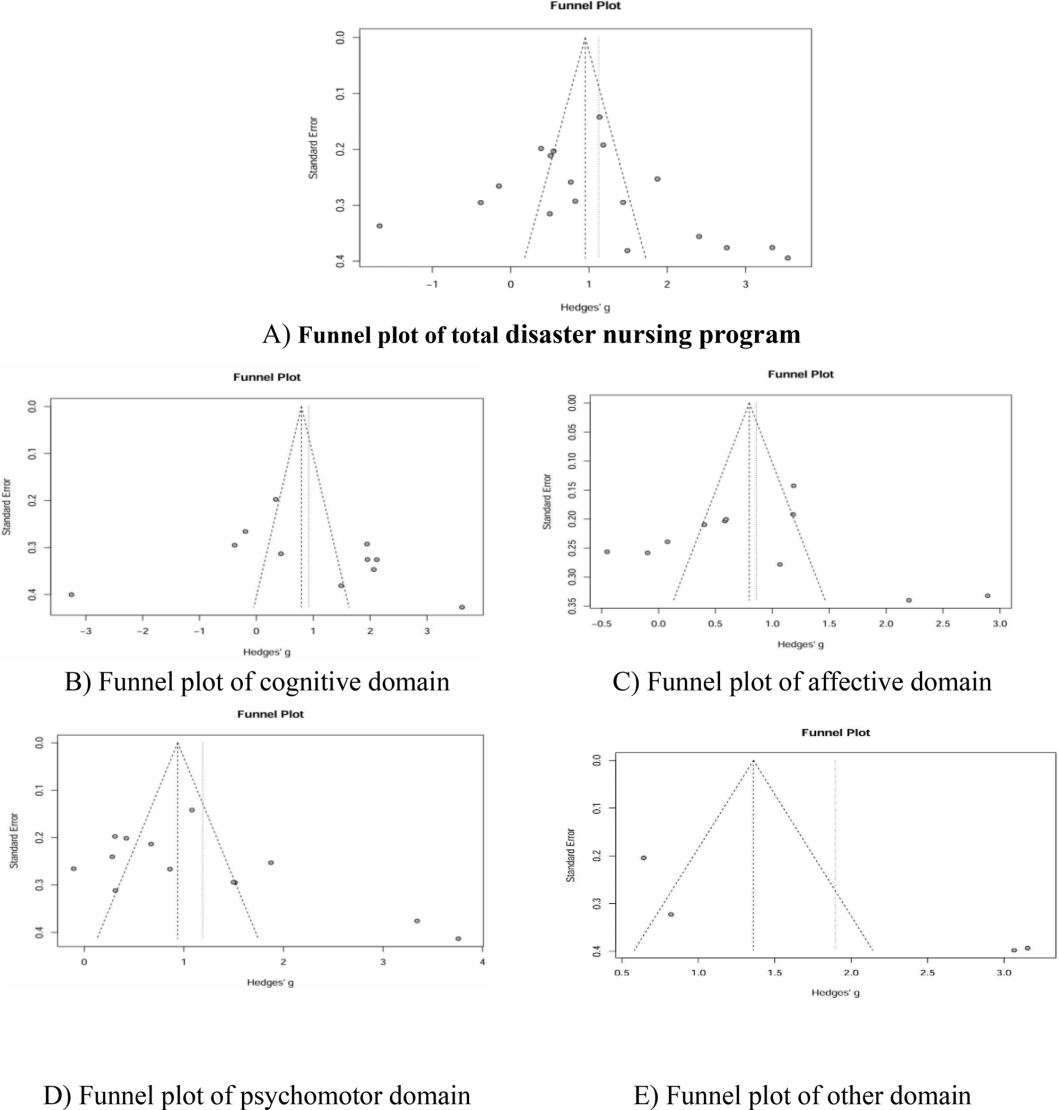
Funnel plot and Trim-and-Fill analysis of publication bias

Publication bias was assessed using both visual and statistical approaches to ensure the robustness of the findings. Visual inspection of the overall funnel plot (Fig. 4) revealed no evident asymmetry, and Egger’s regression test confirmed the absence of significant bias. The fail-safe N was 1,790, substantially exceeding Rosenthal’s criterion of 5k + 10 (k = 18, threshold = 100), indicating that the findings were highly robust [22]. The Trim-and-Fill method suggested the potential absence of two studies; after adjustment, the pooled effect size slightly decreased to Hedges’ g = 0.84 (95% CI [0.13, 1.56]), which remained large and statistically significant.

Cognitive domain: Visual inspection of the funnel plot demonstrated reasonable symmetry. Egger’s regression test (z = 0.69, *p* = 0.509) and Begg’s rank correlation test (*p* = 0.359) indicated no significant publication bias. The fail-safe N was 332, exceeding the robustness criterion of 90 (5k + 10, k = 16). Trim-and-Fill adjustment suggested several potentially missing studies, yielding an adjusted effect size of Hedges’ g = 0.50 (95% CI [−0.69, 1.69]).

Affective domain: The funnel plot exhibited a symmetrical distribution. Statistical tests strongly supported the absence of publication bias, with Egger’s regression test (z = 0.29, *p* = 0.781) and Begg’s rank correlation test (*p* = 0.761) both nonsignificant. The fail-safe N was 564, well above the threshold of 65 (5k + 10, k = 11). The Trim-and-Fill method detected no missing studies, and the adjusted effect size remained unchanged (Hedges’ g = 0.86, 95% CI [0.21, 1.51]).

Psychomotor domain: The funnel plot displayed some scatter but no clear asymmetry. Egger’s regression test was nonsignificant (z = 1.76, *p* = 0.106), although Begg’s rank correlation test approached significance (*p* = 0.042), suggesting the possibility of small-study effects. The fail-safe N was 1,108, far exceeding the threshold of 75, indicating overall robustness. The Trim-and-Fill method detected no missing studies, and the effect size remained consistent (Hedges’ g = 1.19, 95% CI [0.49, 1.89]).

Other integrated domain: Visual inspection revealed slight asymmetry in the funnel plot; however, Egger’s regression test (*p* = 0.128) and Begg’s rank correlation test (*p* = 0.333) were not statistically significant. The fail-safe N was 166, exceeding the robustness threshold of 30. Trim-and-Fill analysis suggested potential missing studies, yielding an adjusted effect size of Hedges’ g = 1.33 (95% CI [−0.86, 3.52]).

The total domain funnel plot showed a reasonably symmetrical distribution across all 18 studies (Egger’s *p* = 0.315, Begg’s *p* = 0.131), with two studies identified by Trim-and-Fill as potentially missing.

The cognitive domain funnel plot exhibited minor asymmetry, consistent with Trim-and-Fill adjustments (Egger’s *p* = 0.509, Begg’s *p* = 0.359). The affective domain plot displayed clear symmetry, with no evidence of bias (Egger’s *p* = 0.781, Begg’s *p* = 0.761).

The psychomotor domain plot showed some dispersion, with Begg’s test (*p* = 0.042) suggesting possible small-study effects (Egger’s *p* = 0.106). The integrated domain plot demonstrated slight visual asymmetry, though statistical tests non-significant (Egger’s *p* = 0.128, Begg’s *p* = 0.333).

Each plot displayed Hedges’ g values on the x-axis and standard error on the y-axis (inverted), with the dotted line representing the expected distribution under the assumption of no publication bias. All plots are available in the online supplementary materials.

Standard error values varied considerably across included studies, reflecting differences in sample sizes and study precision. Studies with larger standard errors (indicative of smaller sample sizes) showed greater variability in effect sizes—a common occurrence in meta-analyses. This variability contributes to the observed heterogeneity and underscores the importance of interpreting findings in light of study quality and precision. The inclusion of several small-sample studies highlights the need for future large-scale, adequately powered research to yield more precise estimates of effect in disaster nursing education.

Overall, the assessment of publication bias across all domains indicated that bias did not pose a substantial threat to the validity of the findings. However, the marginal Begg’s test result for the psychomotor domain (*p* = 0.042) warrants cautious interpretation and emphasizes the necessity for additional high-quality studies in this domain (Fig. 4).

## Discussion

This meta-analysis systematically evaluated the effectiveness of disaster nursing programs by categorizing educational outcomes according to Bloom’s taxonomy, thereby providing an evidence-based foundation for the development of structured curricula in disaster nursing education. From a total of 12,076 domestic and international studies retrieved up to August 6, 2024, 18 met the inclusion criteria, comprising eight RCTs and ten non-randomized controlled trials (non-RCTs). Fourteen studies targeted nursing students, while four involved practicing nurses. The included programs employed various instructional formats, including simulation-based education (*n* = 6), lecture-based education (*n* = 4), and tabletop exercises (*n* = 3), with the majority delivered through face-to-face instruction (*n* = 11).

The pooled effect size of disaster nursing programs was large (Hedges’ g = 1.12). Although direct comparisons across studies are limited by program heterogeneity, this magnitude is comparable to prior meta-analytic findings of patient simulation in nursing education, which reported a medium-to-large effect size (g = 0.71) [44]. The predominance of simulation-based programs that integrate theoretical and practical components likely contributed to these substantial effects. Among the three learning domains, the psychomotor domain demonstrated the largest effect size (Hedges’ g = 1.19), indicating that disaster nursing education is particularly effective in enhancing practical performance. This aligns with previous meta-analyses of scenario-based simulation programs, which showed similarly large effects for clinical skill acquisition (g = 1.45) [45]. Simulation and tabletop exercises, in particular, appear crucial for strengthening essential disaster response competencies, such as triage, emergency care, and situational decision-making. Given the unpredictable and high-stakes nature of disaster contexts, repeated hands-on practice and skill mastery are indispensable for preparedness and effective real-world response.

The cognitive domain produced an effect size of 0.92, which did not reach statistical significance (*p* = 0.139). This contrasts with previous meta-analyses of scenario-based simulation programs reporting moderate gains in knowledge (g = 0.66). The nonsignificant result may reflect limitations in the sensitivity and specificity of existing knowledge assessment instruments [46] and the predominant focus of many programs on experiential rather than theoretical learning. Future research aiming to strengthen cognitive outcomes may benefit from integrating structured theory-based content or blended learning approaches that combine conceptual instruction with experiential application. The affective domain demonstrated a large and statistically significant effect (Hedges’ g = 0.86), suggesting that disaster nursing education effectively fosters self-efficacy, confidence, and positive attitudes toward disaster preparedness and response. These findings underscore the importance of the affective dimension in shaping professional readiness. Attitudinal and motivational changes are more likely to develop through repeated and sustained exposure rather than through isolated training sessions [47]. Notably, most programs included in this review were single-session interventions, highlighting the potential value of longitudinal or reinforcement-based educational designs to further enhance affective outcomes.

The relative magnitudes of effect sizes across domains—other (integrated), psychomotor, cognitive, and affective—were consistent with patterns reported in meta-analyses of problem-based learning (PBL) in nursing education [48]. However, some subgroup analyses of simulation-based nursing education stratified by fidelity level have yielded alternative orderings, with psychomotor, affective, and cognitive domains ranked sequentially [49]. This discrepancy may reflect the greater emphasis on reasoning, adaptability, and problem-solving in PBL and disaster nursing programs compared with the skill-centric focus typical of traditional clinical training.

The findings of this meta-analysis hold important implications for both nursing education and disaster management policy. The large effect observed in the psychomotor domain indicates that current disaster nursing programs effectively enhance practical skills, whereas the non-significant effect in the cognitive domain highlights the need to reinforce theoretical components. Developing effective disaster nursing education requires a balanced, multidimensional approach that integrates strengthened cognitive instruction through case-based discussions and problem-solving activities; longitudinal affective development through multi-session programs incorporating reflection and mentoring; and comprehensive competency assessments encompassing all domains. These findings further support the implementation of the Sendai Framework for Disaster Risk Reduction 2015–2030 [6]—particularly Priority 3, which emphasizes investing in disaster risk reduction for resilience—and align with the WHO Health Emergency and Disaster Risk Management (Health EDRM) Framework [2], which underscores the systematic capacity strengthening of the health workforce. Accordingly, to guide disaster nursing policy, these results should be utilized to establish competency standards, strategically allocate resources to address deficiencies in cognitive domain outcomes, mandate ongoing education and retraining, and link educational performance metrics to measurable disaster response outcomes.

Overall, this study provides a comprehensive and systematic analysis of the effectiveness of disaster nursing programs based on Bloom’s taxonomy, offering essential insights for evidence-based curriculum development. The findings underscore the importance of adopting an integrated educational approach across cognitive, affective, and psychomotor domains, particularly within practice-oriented training programs. However, several limitations warrant consideration. First, this review was restricted to studies published in English and Korean and sourced from a limited number of databases, which may have introduced language and selection bias. Consequently, relevant studies indexed in other databases or published in additional languages may have been excluded, potentially limiting the comprehensiveness of the evidence base. Future systematic reviews should incorporate a broader range of databases and multilingual sources to achieve a more globally representative understanding of disaster nursing education. Second, the number of studies that independently evaluated the psychomotor domain was limited, constraining the ability to generalize findings related to practical skill acquisition. Further research employing standardized assessment tools and consistent methodologies is recommended to validate these findings. Third, most included studies assessed outcomes over the short term, precluding conclusions regarding sustained learning or long-term behavioral changes. Future investigations should therefore prioritize the development of standardized, longitudinal disaster nursing programs, evaluate their long-term impact, and examine how acquired competencies translate to real-world disaster response performance. Additionally, research exploring factors influencing knowledge retention, skill transfer, and sustained competency development will be critical for advancing the field and ensuring the readiness of the nursing workforce in disaster contexts.

## Conclusions

This meta-analysis evaluated the effects of disaster nursing programs on nurses and nursing students through the framework of Bloom’s taxonomy. Data synthesized from studies published up to August 6, 2024, demonstrated a large overall pooled effect size (Hedges’ g = 1.12). Domain-specific analyses revealed effect sizes of Hedges’ g = 1.19 for the psychomotor domain, 0.92 for the cognitive domain, 0.86 for the affective domain, and 0.89 for other integrated domains. Notably, the psychomotor estimate was derived from a single study and should therefore be interpreted with caution.

The findings indicate that disaster nursing programs are predominantly practice- and skill-oriented, particularly through modalities such as simulation-based learning and clinical procedure training. To further enhance program effectiveness, educational strategies should aim to reinforce theoretical comprehension and facilitate the internalization of professional values and attitudes. By systematically evaluating program outcomes within the structure of Bloom’s taxonomy, this study provides foundational evidence to inform the development of balanced, evidence-based disaster nursing curricula. Future program design should emphasize integration across cognitive, affective, and psychomotor domains, ensuring that learning outcomes are clearly aligned with these educational objectives to cultivate comprehensive competency in disaster preparedness and response.

## Data Availability

The datasets generated and/or analyzed during the current study are not publicly available but are available from the corresponding author upon reasonable request.

## Abbreviations

AD: Affective domain
BGDPS: Belief in General Disaster Preparedness Scale
BRS: Brief Resilience Scale
CD: Cognitive domain
CI: Confidence interval
DPPSN: Disaster Preparedness Perception Scale for Nurses
DRSES: Disaster Response Self-Efficacy Scale
Health EDRM: Health Emergency and Disaster Risk Management
Hedges’ g: Effect size metric
I^2^: Percentage of variation due to heterogeneity
ICN: International Council of Nurses
k: Number of studies
LMS: Learning Management System
N/A: Not available
non-RCT: Non-randomized controlled trial
O: Others (knowledge, attitude, skills)
PBL: Problem-based learning
PD: Psychomotor domain
PRISMA: Preferred Reporting Items for Systematic Reviews and Meta-Analyses
Qb: Q-value between subgroups
R: R statistical software
RCT: Randomized controlled trial
RoB 2.0: Risk of Bias tool 2.0
ROBINS-I: Risk of Bias in Non-randomized Studies of Interventions
UNDRR: United Nations Office for Disaster Risk Reduction
USD: United States dollar
WHO: World Health Organization.
